# The effects of exercise and ambient temperature on dietary intake, appetite sensation, and appetite regulating hormone concentrations

**DOI:** 10.1186/s12986-019-0348-5

**Published:** 2019-05-06

**Authors:** Iva Mandic, Mavra Ahmed, Shawn Rhind, Len Goodman, Mary L’Abbe, Ira Jacobs

**Affiliations:** 1 Faculty of Kinesiology and Physical Education, University of Toronto, 55 Harbord Street, Toronto, ON M5S 2W6 Canada; 2 Department of Nutritional Sciences, University of Toronto, Toronto, ON M5S 3E2 Canada; 3 Defence Research and Development Canada, Toronto Research Centre, Toronto, ON M3K 2C9 Canada

**Keywords:** Appetite, Exercise, PYY, GLP-1, Acylated ghrelin, Leptin, Dietary intake

## Abstract

**Background:**

It is not clear whether the frequently reported phenomenon of exercise-induced anorexia is exacerbated or blunted in warm or cold environments. Therefore, this study investigated the effects of exercise in three different environmental temperatures vs. rest, on perceptions of appetite, appetite regulating hormones, and food intake.

**Methods:**

In a randomized repeated-measures design, 18 Canadian Armed Forces members (14 male, 4 female) completed four 8-h trials in a thermally-controlled chamber: one 8-h resting trial at 21 °C (Sedentary); and three trials where participants completed two 2-h circuits of standardized military tasks interspersed with two 2-h rest periods, once at 30 °C (Hot), once at 21 °C (Temperate), and once at − 10 °C (Cold). Participants consumed military field rations ad libitum and had their appetite assessed with visual analogue scales. Plasma concentrations of GLP-1, PYY, acylated ghrelin, and leptin were also determined.

**Results:**

Appetite was perceived as being suppressed in the heat compared to the cold (*p* < 0.05). While neither exercise nor environmental temperature altered circulating GLP-1 levels, exercise in all environments increased blood concentrations of PYY (*p* < 0.05). Leptin concentrations were elevated in the heat and diminished in the cold (p < 0.05), and acylated ghrelin concentrations were affected by both exercise and ambient temperature resulting in Sedentary = Cold>Temperate = Hot (*p* < 0.05). Contrary to the changes in appetite perceptions and hormonal concentrations, dietary intake was not different between conditions (*p* > 0.05). Relative energy intake (total 24 h energy intake minus 24 h energy expenditure) on the other hand, was significantly higher during the Sedentary condition than it was during any of the active conditions (p < 0.05). Most (83%) of the participants were in a positive energy balance during the Sedentary condition, whereas during most (80%) of the active conditions (Hot, Temperate, Cold) participants were in a negative energy balance.

**Conclusions:**

In this study where food was freely available, variations in ambient temperature, exercise vs. rest, appetite-regulating hormone concentrations, and subjective appetite sensation were not associated with any changes in dietary intake within 24-h of acute, prolonged exercise.

## Introduction

Several studies have assessed the acute effects of exercise on appetite, appetite-regulating hormone concentrations, and energy intake (EI) [15, 17, 36, 44, 46, 49, 51, 66, 67, 69]. This literature indicates that acute exercise typically shifts the hormonal milieu towards appetite suppression during and for about 30 min following exercise and is associated with variations in several appetite regulating hormones. While hormonal changes are not always detected following acute exercise, decreases in the concentrations of the appetite stimulating hormone acylated ghrelin [15, 17, 36, 44, 46, 69] and increases in various appetite suppressing hormones including peptide YY (PYY) [36, 51, 66, 67] and glucagon-like peptide-1 (GLP-1) [36, 51, 66, 67] are usually found. These hormonal shifts have been reported in association with a decrease in subjective appetite [17, 49], although not always [15, 19]. Interestingly, this “appetite suppressing” hormonal response is typically not followed by a subsequent change in EI [10, 69]. Some studies reported a decrease in EI [66, 67], while others reported minor increases in EI [51, 60]. Regardless, relative energy intake (REI) (which is equivalent to total EI minus total energy expenditure) decreases as a result of exercise causing a short term negative energy balance [58].

It is thought that environmental temperature might also affect appetite, as the results of early cross-sectional military research suggests that food intake is low in the heat and high in cold environments [42]. In human laboratory studies, acute (≤48 h) changes in ambient temperature 31 °C vs. 22 °C [28], 27 °C vs. 22 °C [72], ~ 19.5 °C vs. ~ 26.5 °C [11], 16 °C vs. 22 °C [73], or 18 °C vs. 24 °C [47] did not significantly impact subjective appetite or EI, although in some cases a trend in the expected direction appeared [72]. Few studies have examined the effect of ambient temperature without exercise on appetite-regulating hormones. Acute (30–100 min) cold (2–6 °C) exposure has previously been reported to decrease plasma leptin [54, 56], and increase plasma ghrelin levels [62]. Whereas acute (30-100 min) heat (30–31 °C) exposure has been reported to decrease plasma ghrelin levels [28, 62]. It is unclear how the interaction between exercise and ambient temperature impacts appetite and EI. Some studies suggest that appetite is reduced and the hormonal responses associated with appetite suppression are augmented with higher ambient temperature [60], while the inverse was seen with lower ambient temperature [22]; other studies are equivocal [46]. To our knowledge, only one study [46] has reported the impact of exercise in all three (hot, temperate and cold) ambient temperatures on appetite in the same participants, but in that study actual food intake was not assessed.

An understanding of how exercise in varying temperatures affects appetite and EI can be important for those who participate in outdoor sports, in occupational, and military operational settings requiring prolonged outdoor physical exertion. In military personnel for example, body weight loss due to under-eating during field training and deployed operations is not uncommon even when food is plentiful [6, 7, 27, 32, 35, 39, 61]. This ‘voluntary anorexia’ can at times result in rapid weight loss that can exceed 10% of initial body weight and lead to immune function suppression, and decreased physical and cognitive performance [53, 59]. Although this ‘voluntary anorexia’ is often explained by disliked food palatability, insufficient time to eat, and inconvenient or lengthy food preparation [9, 30, 50], it is possible that voluntary anorexia in military personnel is also mediated by hormonal responses.

Therefore, the purpose of this study was to investigate the effects of arduous physical activity in varying ambient temperatures on dietary intake, appetite sensation, and appetite-regulating hormone concentrations in military personnel throughout an eight hour day.

## Methods

### Participants

Twenty-seven healthy, male (*n* = 21) and female (*n* = 6) Canadian Armed Forces (CAF) members volunteered to participate in the study (Table 1). Participants were either Regular Force CAF or Class A reservists. Seven withdrew voluntarily after starting the study due to time constraints (*n* = 3), reconsideration (n = 3), or loss of contact (*n* = 1). Two participants were also excluded due to their non-compliance with the study restrictions. As a result, 18 participants (14 male, and 4 female) completed the study. All participants were free from metabolic and cardiac disorders and were not taking any medications or nutritional supplements. All participants were fully informed of the details, discomforts and risks associated with the experimental protocol before being asked for their written informed consent. Informed consent was obtained from all individual participants included in the study. The study protocol was reviewed and approved by institutional human research ethics committees at Defence Research and Development Canada (#2013–075), and the University of Toronto (#29914).

**Table 1:** Participant characteristics

Participant Status	Sex	Age (y)	Height (cm)	Body Mass (kg)	BMI	Body Fat (%)	*V̇O*_2Peak_ (mL·min^− 1^·kg^− 1^)
Completed	14 males 4 females	33.5 ± 10.8	173.8 ± 10.3	80.4 ± 15.7	26.5 ± 4.0	23 ± 8	43.8 ± 6.1
Withdrawn	7 males 2 females	28.9 ± 6.4	171.0 ± 9.7	75.3 ± 8.8	25.8 ± 3.4	21 ± 9	43.7 ± 6.2
All	21 males 6 females	32.0 ± 9.7	172.8 ± 10.0	78.7 ± 13.8	26.3 ± 3.6	22 ± 8	43.8 ± 6.0

There were no significant differences between individuals who completed the study (completed), and those who did not (withdrawn). Data are presented as means ± SD

#### Experimental design

**Fig. 1:**
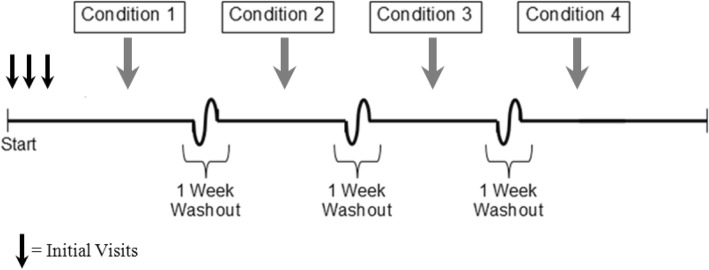
Graphical depiction of the study design. All study participants (*n* = 18) underwent each condition, and the order of these conditions was assigned in a randomized manner using a computerized number generator https://www.random.org/

The study design is graphically depicted in Fig. 1**.** Participants made three initial visits to the lab, followed by an additional four for the experimental conditions.

### Initial visits

Visit 1 consisted of the completion of the informed consent form, PAR-Q+ to clarify if health risks suggested precluding their engagement in arduous exercise [70], and the Pittsburgh Sleep Quality Index (PSQI) [8]. During Visit 2, participants had their peak aerobic power (*V̇O*_*2peak*_) measured using indirect calorimetry during a progressive incremental exercise test to voluntary exhaustion on a treadmill [18]. Oxygen consumption was measured and averaged every 30 s; the highest value that was obtained was defined as the *V̇O*_*2peak*_. During Visit 3, participants reported to the laboratory after a 10 h overnight fast, after having completed a three-day weighed food record to assess each participant’s typical dietary intake. Body mass was measured with a standard scale (HealthOMeter Continental Scale Corp., Bridgeview IL, USA), and percent body fat was estimated via air-displacement plethysmography (BOD POD™, COSMED, Rome, Italy). Measurements were completed two consecutive times to confirm accuracy.

### Experimental conditions

Participants completed a total of four different experimental conditions; each condition started in the morning and lasted 8 h in the environmental chamber. At least one week intervened between conditions (Fig. 1). For the two days prior to each condition, participants were given military field rations to consume at home (1 breakfast, 1 lunch, and 1 dinner ration per day was provided). Participants ate rations for two days leading up to each condition for three reasons: 1) in order to more accurately mimic field conditions where CAF would consume military rations exclusively for several days or weeks at a time; 2) in order for participants to become accustomed to eating military rations and prevent participants from changing their eating behaviour due to the palatability or novelty of the provided food (in the event that military rations are disliked for example, participants would more easily be able to modify their intake for a single day, as opposed to three days consecutively i.e. over-eat prior to the experimental day in anticipation of receiving military rations and undereating on the experimental day); and 3) to determine if the laboratory environment itself affected food intake (this was done by comparing military ration intake at home with military ration intake in the Sedentary condition). Participants were asked to document their food intake, consume ad libitum only the provided military rations and water, and bring back all unconsumed and partially eaten items. Participants were required to refrain from physical activities (above activities of daily living) for the two days prior to each condition, and for the remainder of the study day after leaving the environmental chamber. In addition, participants were asked to refrain from ingesting food or water after 10 pm the night prior to each experimental condition. There were four experimental conditions: participants completed one sedentary condition (Sedentary) where they rested for 8 h in the environmental chamber where ambient temperature was controlled at 21.1 ± 0.3 °C, and the relative humidity (RH) was maintained at 29 ± 2%. Participants also completed three active conditions where they executed two 2-h circuits composed of a standardized set of typical military tasks with 2-h rest following each circuit. Each of these three active conditions was identical except that the maintained temperature in the environmental chamber was different so that the protocol was completed: once at 30.1 ± 0.2 °C, RH 31 ± 1% (Hot), once at 21.0 ± 0.2 °C, RH 32 ± 4% (Temperate), and once at − 10.4 ± 0.4 °C, RH 56 ± 3% (Cold). All of the participants underwent every condition and the order of these conditions was assigned in a randomized and counter balanced manner. There were 24 possible condition orders, and a computerized number generator (https://www.random.org/) determined which permutation a participant would be assigned to.

### Order of events during each experimental condition (Fig. 2)

At 6:00 am on the morning of each experimental condition, participants swallowed a telemetric temperature capsule for monitoring of internal body temperature with 250 mL of water at home. At 7:45 am, participants reported to the laboratory for initial measurements of body weight measured using a standard scale (HealthOMeter Continental Scale Corp., Bridgeview IL, USA), resting blood pressure using an automated blood pressure cuff (Omron Healthcare, Kyoto, Japan), and core temperature using the Equivital™ (Hidalgo, Cambridge, United Kingdom). The visual analogue scales for appetite (VASA) (described in the measurements section) were also administered while fasting. Participants consumed breakfast, and then donned the temperature appropriate military clothing for the environmental condition on that day. Participants wore standardized military clothing items that are mandatory during field operations; these included: military fatigues, a tactical vest (~ 3-7 kg), a fragmentation protection vest (2-3 kg), combat boots (1.5 kg- 2.5 kg), and a helmet (~ 1.5 kg). Other than these required clothing, participants decided which military clothing to wear or not wear in order to be comfortable in the condition that they would be enduring (gloves, long underwear, coat, etc.). Participants also wore a portable metabolic system: the Metamax 3B (CORTEX Biophysik GmbH, Leipzig, Germany), which weighed about 1.5 kg. This device required the participant to wear an oral-nasal mask over the face and an interface box strapped to the torso. A heart rate strap (Polar, Kempele, Finland), that was compatible with the Metamax 3B unit, was also affixed to the participant’s chest, as was a device for the monitoring of internal body temperature from a telemetered ingestible temperature capsule (Equivital™, Hidalgo, Cambridge, United Kingdom). The participants then entered the environmental chamber in which room temperature and relative humidity were controlled for the day’s duration. The environmental chamber was 5.9 m long and 4.5 m wide.

**Fig. 2:**
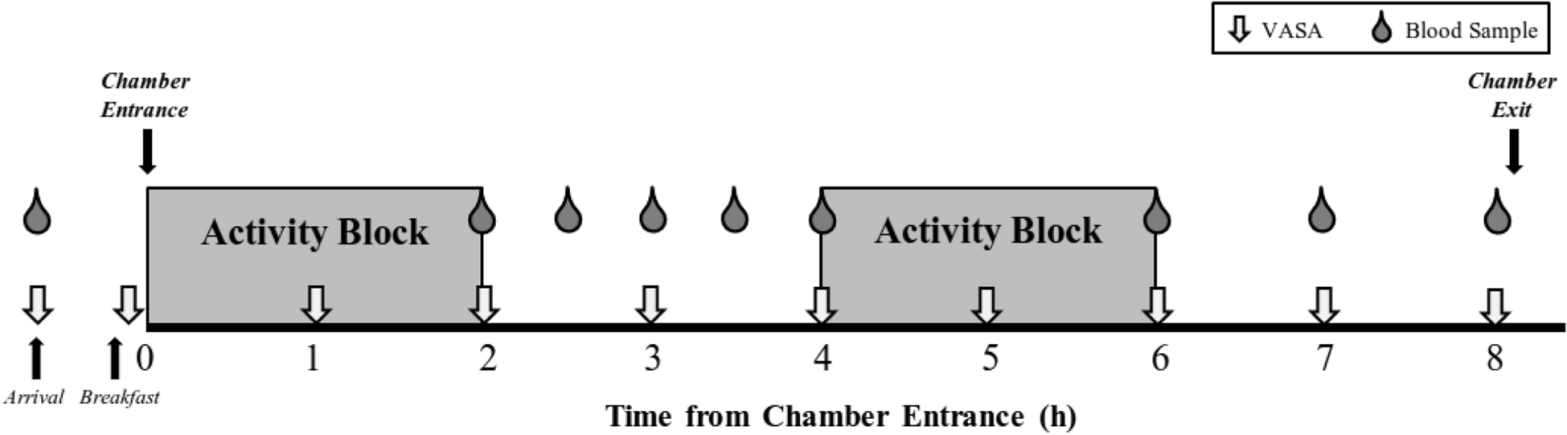
Schematic of each experimental condition. Grey boxes represent the two-hour ‘activity blocks’, although during the Sedentary condition the participants were inactive during these blocks. Participants arrived to the laboratory following a 10 h overnight fast; the first visual analogue scale for appetite (VASA), as well as the first blood sample were collected upon arrival. Fasting and Post-Breakfast data points were collected outside of the environmental chamber prior to trial commencement. The 8-h trial began once the participant entered the environmental chamber; this occurred within minutes of the participants completing their breakfast

Following entry into the chamber, participants either rested (Sedentary) or they completed the standardized military tasks (Cold, Temperate, and Hot). The infantry activities completed during the circuits included resting activities (sitting, kneeling, standing, lying down), and both aerobic (treadmill walking at various speeds (2.4 km/h, 4 km/h, 5.6 km/h), inclines (0% grade, 5% grade, 10% grade), and while carrying different loads (no load, 10 kg day pack, 20 kg rucksack)) and strength / muscular endurance (moving sandbags (20 kg each), varying ammo cans (3.2 kg and 13.7 kg) and jerry cans (2.5 kg each, and 20 kg each), dragging an 85 kg dummy across the floor, building a sandbag barrier, etc.) activities. When classifying the intensity of the activities based on the percentage of maximal heart rate (ACSM Classification of Physical Activity Intensity [5] participants were found to be engaged in *very light* activity (< 50% max HR) 7% of the time, *light* activity (50 to < 64% max HR) 29% of the time, *moderate* activity (64 to < 77% max HR) 31% of the time, *vigorous* activity (77 to < 94% max HR) 26% of the time, and *very vigorous* (> 94% max HR) 6% of the time. After 8 h, participants exited the environmental chamber and took their supper ration – and any leftover items from their breakfast and lunch rations – home with them. Participants continued to document all food that was eaten upon leaving the laboratory and returned all waste and uneaten food the following day.

#### Measurements

##### Sleep quality

Sleep quality was assessed with questionnaires in order to better understand the sleep patterns of the participants since studies have shown that food intake is affected by sleep deprivation [14]. Both the PSQI, −which assesses sleep over the last month (conducted once during Visit 1) [8], and the Groningen Sleep Quality Scale (GSQS)[52], −which assesses how a participant slept the night before their visit (conducted the morning of each chamber day) were used.

##### Energy expenditure (EE)

Four hour EE (as calculated using Acheson’s RQ-based equation [1]) was assessed continuously based on the respiratory exchange data collected with the portable oxygen uptake measurement system. The system was worn during the entire activity portions (4 h) on each chamber day and participants only removed the respiratory mask to drink water or, on rare occasions consume food. Twenty-four hour EE was also estimated assuming that participants slept for eight hours and that they expended 0.95 METs while asleep [4]. Additionally, since participants were required to (and confirmed that they had) abstain from exercise for the two days prior to and on the chamber day, it was presumed that for the remaining 12 waking hours while EE was not measured, that participants expended energy at the same rate that they had during the Sedentary condition. As a result 24 h EE was calculated as follows:

*Where EE = energy expenditure, H=Hot condition, C=Cold condition, T = Temperate condition, S=Sedentary condition, BM = body mass in kg, and where 1 MET is equivalent to 1 kcal/kg/h *[4].

##### Dietary intake

There were 18 different military field rations choices available to the participants (6 breakfast, 6 lunch, 6 dinner), and the participants selected 1 breakfast, 1 lunch, and 1 dinner ration which was kept the same for all of the chamber days (Hot, Temperate, Cold, Sedentary) for that participant. The three rations (breakfast, lunch and dinner) provided 4093 ± 263 kcal and approximately 627 g of carbohydrate, 191 g of fat, and 108 g of protein. Each ration contained an entrée, dessert, sport drinks, bread, jam/peanut butter/honey, two hot beverages, condiments and depending on the ration, additional products such as chocolate bars and candy. In addition to the three rations, participants could take one snack ration pack (1125 kcal) containing energy bars, sports drinks and other snack foods. Although military rations are provided as separate meals, items from any of these packs could be (and often are) consumed at any time. To determine the timing of food intake, the 8-h in the environmental chamber was segmented into 30 min intervals (0–0.5 h, 0.5–1 h etc.) to determine when participants chose to eat. Investigators documented all feeding behaviours (selection, amount, and timing of food intake) and weighed all food that was discarded. The CAF Directorate of Food Services provided (from the manufacturer) the nutritional information for all of the presented rations, some of which were based on chemical analyses. Dietary intake (EI and macronutrient intake) was determined by subtracting the energy and macronutrient content of the unconsumed food from the known quantities in the rations provided. Relative energy intake (REI) was calculated by subtracting estimated 24 h EE from the energy consumed (kcal) throughout the day. From REI, it was determined whether a participant was in a positive or negative 24-h energy balance.

##### Subjective appetite sensation

Since visual analogue scales have been found to be reliable, relatively accurate, and sensitive especially when used in repeated measures designs [29], ten-cm VASA were used to assess appetite sensation. Four indices were assessed: *hunger*, *satiety*, *fullness* and *prospective eating*. Subjective appetite was assessed while fasting, immediately following breakfast, and every hour throughout the 8 h in the environmental chamber (Fig. 2).

#### Blood sampling

##### Blood sample timing

During each experimental condition, venous blood samples were drawn from an antecubital vein using an indwelling catheter which was kept patent via infusion of saline (0.9% sodium chloride). Blood samples were taken immediately upon arrival to the laboratory (time point 0), at the end of the first exercise bout (beginning of the first rest period) (time point 2 h), 30, 60, and 90 min into the first rest period (time point 2.5 h, 3 h, and 3.5 h respectively), 120 min into the first rest period (immediately before the second exercise bout) (time point 4 h), at the end of the second exercise bout (beginning of the second rest period) (time point 6 h), 60 and 120 min into the second rest period (time point 7 h and 8 h respectively) while in the chamber (Fig. 2).

##### Collection procedures

Four (4) mL were taken at each blood sample time point and assayed for the plasma concentrations of leptin, acylated ghrelin, GLP-1, and PYY. Samples for the determination of GLP-1, PYY, and leptin were collected in a chilled 2 mL K_2_EDTA blood collection tube that was injected with a 167uL aprotinin solution containing 0.167 mg of aprotinin (500KIU/mL blood) and 20uL of DPP-IV inhibitor (Millipore, Darmstadt, Germany). Samples for the determination of acylated ghrelin were collected in a chilled 2 mL K_2_EDTA blood collection tube that was injected with an 80uL 4-(2-Aminoethyl) benzenesulfonyl fluoride hydrochloride (AEBSF) solution containing 2 mg of AEBSF [12]. Once drawn, blood samples were centrifuged immediately at 1000 x g for 15 min at 4 °C, and frozen at − 70 °C until subsequent biochemical analysis. An additional three (3) mL was collected and analyzed immediately for the measurement of haemoglobin and hematocrit, in order to standardize for plasma volume changes [24].

##### Blood analysis

The concentrations of leptin, GLP-1, PYY, and acylated ghrelin in the blood samples were determined using electrochemiluminescent immunoassays (ECLIA). Three different Meso Scale Diagnostics (MSD) assays were run, one for the determination of GLP-1 and leptin (Custom Human Metabolic Duplex - N45ZA-1, Rockville, Maryland), one for the determination of PYY (Human Total PYY - K151MPD-2, Rockville, Maryland), and one for the determination of acylated ghrelin (Custom Human Active Ghrelin - N45ZA-1, Rockville, Maryland). The sensitivity of the assays was 0.3 pg/mL for GLP-1, 56 pg/mL for leptin,13 pg/mL for PYY and 9 pg/mL for acylated ghrelin. The inter- and intra-assay coefficients of variation were 12 and 9% for GLP-1, 13 and < 5% for leptin, 8 and 6% for PYY and 14 and 9% for acylated ghrelin respectively. All samples for one participant were run in duplicate on the same assay; as a result the effects of inter-assay variation were controlled within participants.

#### Statistical analyses

Two-factor, repeated measures ANOVAs were used to examine differences between conditions over time for appetite sensation, EE, dietary intake, and REI. Mauchly’s Test was used to test the assumption of sphericity, and where Mauchly’s test was significant, the Greenhouse-Geisser estimate epsilon was assessed. If epsilon< 0.75, then the degrees of freedom were corrected using the Greenhouse-Geisser correction; if epsilon> 0.75, then the degrees of freedom were corrected using the Huynh-Feldt correction [31]. Where significant main effects were found, post hoc analysis was performed using the Bonferroni correction for multiple comparisons. Similar repeated measures ANOVAs (as described above) were also used to examine differences between conditions, and between visit numbers for GSQS scores. Due to the vagaries of blood sampling from intravenous catheters in such experiments in an environmental chamber, several blood samples from different subjects at different time intervals were missed. As a result, appetite-regulating peptide hormone data were compared using linear mixed model analyses of within-subject differences with the fixed effects of condition, time, and the interaction of condition and time. A participant-level random intercept was included to account for the within-subject correlation. Based on the fit statistics of Akaike information criterion (AIC) and Bayesian information criterion (BIC), a compound symmetry covariance structure was found to be optimal in each case. Post hoc analysis was performed using the Bonferroni correction for multiple comparisons to determine statistical significance. Area under the curve (AUC) calculations were made using the trapezoidal method. Pearson product moment correlation coefficients were also used to examine relationships between variables. Data are expressed as mean ± standard deviation (SD) unless otherwise stated. Statistical analyses were carried out using the SPSS v. 22.0 software package, and statistical significance was accepted at *p* < 0.05.

## Results

Dietary intake (both EI and macronutrient intake) for the two days prior to each chamber day was not significantly different between conditions [3], nor was EI different from their habitual intake (which was determined through the 3-day weighed food record completed by participants and submitted on visit 3) (ration intake at home 2688 ± 619 kcal vs. habitual intake 2657 ± 580 *p* > 0.05)[2]. There was no significant difference in how well the participants slept the night before each condition, as the GSQS score did not differ between any of the conditions and there was also no significant effect of visit number (participants did not sleep better or worse due to being in the later stages of the study) (p > 0.05). In addition, the different ambient temperatures employed in the current study varied enough to significantly impact both heart rate and core temperature with Hot>Temperate>Cold *p* < 0.05.

### Energy expenditure (EE)

As expected, the measured EE was significantly lower during the Sedentary condition compared to the others, and approximately 4-times greater during the 4 h of standardized military tasks compared to the same 4 h during the Sedentary condition (p < 0.05). The estimated daily EE was 56–58% greater (p < 0.05) during the different active conditions compared to the Sedentary condition (Table 2). There were no significant differences among the three activity conditions for the 4-h EE or for the estimated 24-h EE (p > 0.05).

**Table 2:** Energy expenditure (EE) and relative energy intake (REI) by condition

	Cold	Hot	Temperate	Sedentary
4-h EE^b^ (kcal)	1705 ± 309	1691 ± 268	1642 ± 320	404 ± 68^a^
Estimated 24-h EE (kcal)	3529 ± 518	3514 ± 499	3465 ± 552	2227 ± 327^a^
REI (Kcal)	− 663.06 ± 959.42	− 432.39 ± 973.52	− 542.07 ± 914.48	+ 696.61 ± 1002.85^a^

^a^denotes significantly different from all other conditions *p* < 0.05

^b^Energy expended during the four hours that the oxygen uptake measurement system was worn

Data are presented as Mean ± SD

### Energy intake (EI) and relative energy intake (REI)

**Fig. 3:**
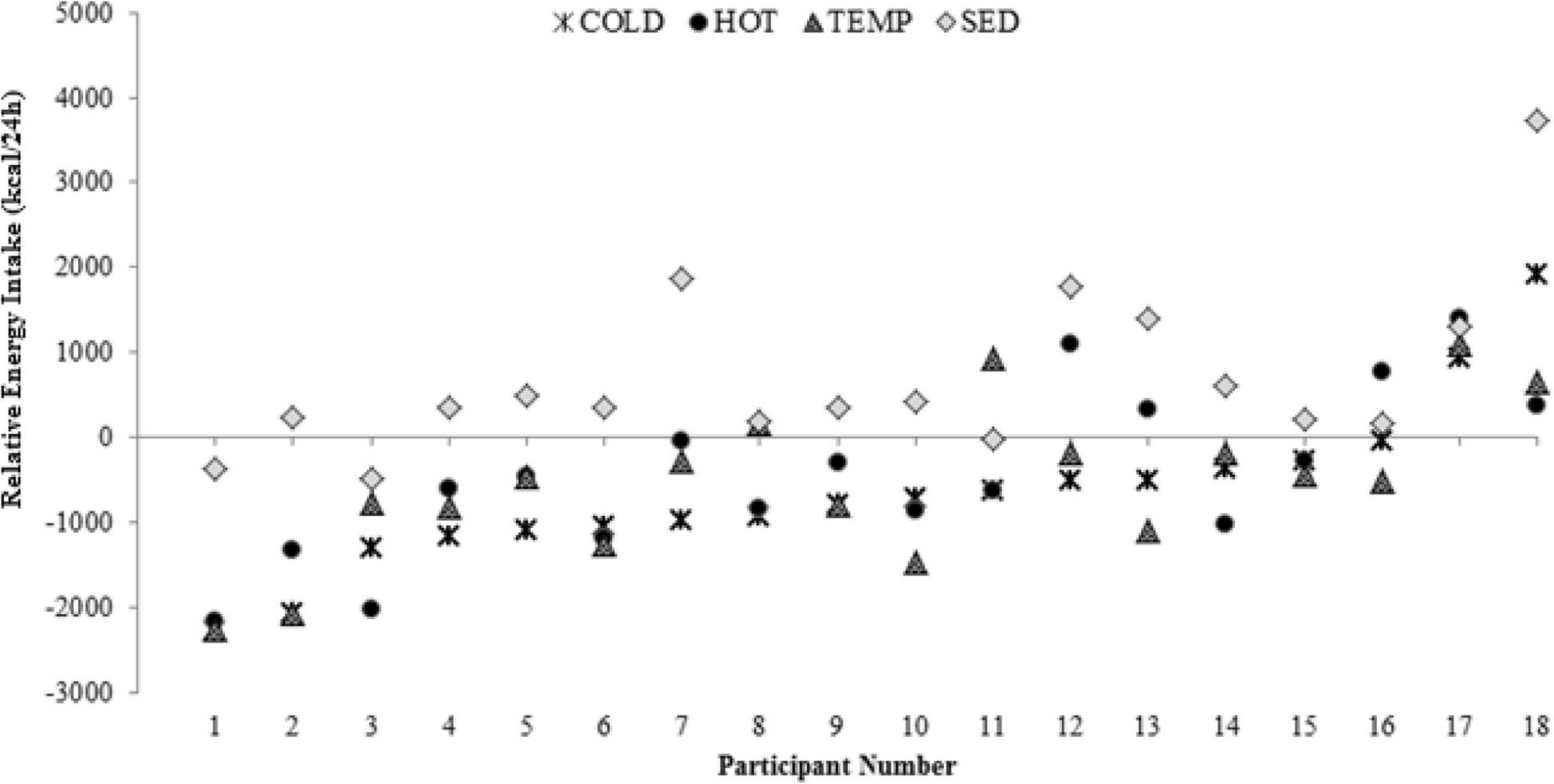
Relative energy intake (REI) for each individual at the end of each trial day (Cold: triple cross; Hot: black circle; Temperate: checkered triangle; Sedentary: grey diamond)

Although 24-h EI was not different between conditions, there was a significant main effect of condition on REI (*p* < 0.05) (detailed analyses on EI are presented elsewhere [3]). REI was significantly higher during the Sedentary condition than it was during any of the active conditions (p < 0.05). No other differences were observed (Table 2). Only three of 18 (17%) participants were in a negative energy balance during the Sedentary condition, whereas participants were in a negative energy balance during 43 out of the 54 (80%) active conditions (Hot, Cold, Temperate). In the Cold condition, only two participants were in a positive energy balance, whereas in the Temperate and Hot conditions four and five participants had a positive energy balance, respectively (Fig. 3).

### Dietary consumption

**Fig. 4:**
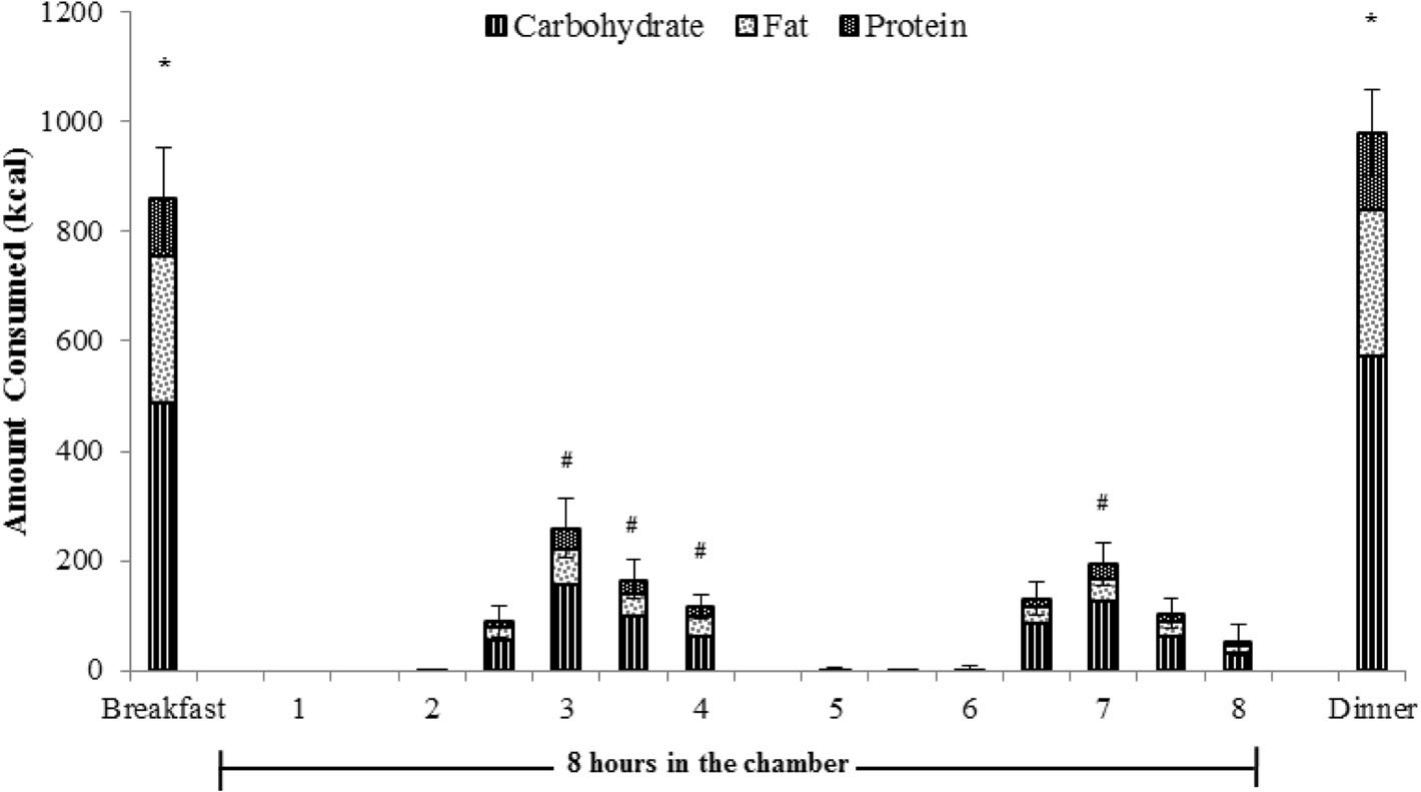
Average amount of total energy (height of total bar) and macronutrients (Carbohydrate: ; Fat: ; Protein: ; portions of the total bar) consumed (in kcal) throughout the experimental days. The two-hour ‘activity blocks’ occurred between 0 and 2 h and between 4 and 6 h, although during the Sedentary condition the participants were inactive during these blocks. During these time periods, participants wore an oxygen uptake measurement system and had their energy expenditure assessed. Breakfast and dinner were consumed outside of the environmental chamber with all other food consumed throughout the 8-h in the chamber. Dietary consumption is displayed in 30 min increments throughout the 8 h inside the chamber. * indicates that amount of energy and macronutrients consumed was significantly different from all time points except for Breakfast and Dinner *p* < 0.05. # indicates that that amount of energy and macronutrients consumed was significantly different from 0 to 0.5 h, 0.5–1 h, 1–1.5 h, and 4–4.5 h p < 0.05. Data are presented as means. SEM is shown for total energy intake only

Detailed analyses on dietary intake are presented elsewhere [3], a brief description of data relevant to appetite are discussed below. There were no significant effects of condition, or interaction with time for the amount of energy or macronutrients consumed; there was however a significant main effect of time on total energy consumed (*p* < 0.05). More energy was consumed at breakfast (856 ± 393 kcal) and dinner (982 ± 330 kcal) than at any of the sixteen 30-min intervals in the chamber (p < 0.05) (Fig. 4). During the activity periods while the portable oxygen uptake measurement device was worn, it was understandably more awkward to eat, and the majority of participants abstained from eating during this time. While in the chamber, participants tended to eat the most about 30 min following each activity period (2.5–3 h post start (261 ± 233 kcal) and 6.5–7 h (192 ± 166 kcal) post start) as compared to other 30-min intervals (*p* < 0.05) **(**Fig. 4). The amount of protein, carbohydrate, and fat consumed throughout the day followed a similar trend to the total energy consumed at each time point **(**Fig. 4). The individual variability of EI from condition to condition was fairly large. In order to decrease the likelihood of a type II error, EI was divided into three categories: 1) typical energy intake (defined as intake within 10% of a participant’s median intake), 2) low energy intake (consumption below 10% of a participant’s median intake), and 3) high energy intake (consumption above 10% of a participant’s median intake). When EI was described in this way, around 50% of the time individuals consumed within 10% of their median caloric intake regardless of the condition, during the remaining conditions, participants’ EIs fluctuated by more than 10% of their median caloric intake (Table 3).

**Table 3:** Percentage of participants whose energy intake fell within, or varied by more than 10% of their median energy intake^a^ in each condition

Condition	Number of participants who consumed MORE than 10% of median intake (%)	Number of participants who consumed within 10% of median intake (%)	Number of participants who consumed LESS than 10% of median intake (%)
Cold	2 (11%)	10 (56%)	6 (33%)
Hot	7 (39%)	8 (44%)	3 (17%)
Temperate	4 (22%)	10 (56%)	4 (22%)
Sedentary	5 (28%)	8 (44%)	5 (28%)

^a^Median energy intake was calculated for each participant using the data from the 8 days (2 days before each condition) that military rations were consumed at home

### Visual analogue scale for appetite (VASA)

#### Time

**Fig. 5:**
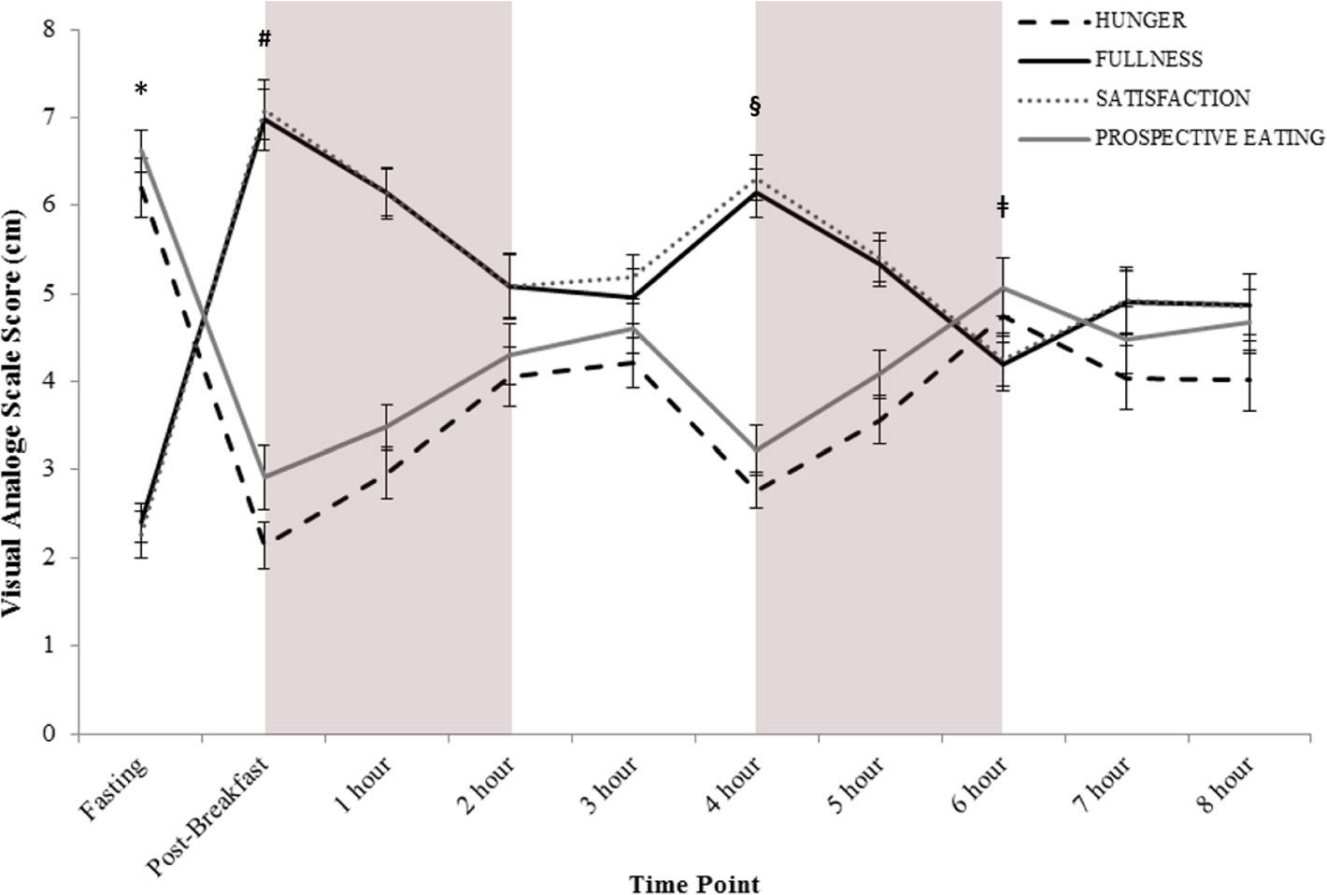
Average appetite scores for all participants, during all of the conditions, as collected by visual analogue scales for the 4 indices of appetite (*hunger* (black line); *fullness* (dark grey white dotted line); *satisfaction* (grey black dotted line); *prospective eating* (grey line)). Grey boxes represent the two-hour ‘activity blocks’, although during the Sedentary condition the participants were inactive during these blocks. Fasting and Post-Breakfast data points were collected outside of the environmental chamber prior to trial commencement. The 8-h trial began once the participant entered the environmental chamber; this occurred within minutes of the participants completing their breakfast. * indicates that all 4 indices were significantly different from all other time points p < 0.05. # indicates that all 4 indices were significantly different from Fasting, 2 h, 3 h, 6 h, 7 h, and 8 h p < 0.05. § indicates that all 4 indices were significantly different from Fasting, 5 h, and 6 h p < 0.05. ǂ indicates significantly different from Fasting, Post-Breakfast, 1 h, 4 h, and 5 h p < 0.05. Data are presented as mean ± SEM

As expected, there was a significant main effect of time on *hunger*, *fullness*, *satisfaction*, and *prospective eating* (*p* < 0.05). *Fullness* and *satisfaction* scores shared a similar trend over time and participants felt the least full and the least satisfied upon arrival to the lab (following a > 10 h overnight fast), and they felt the most full and the most satisfied following breakfast. *Hunger* and *prospective eating* scores on the other hand, while being similar to each other, were contrary to *fullness* and *satisfaction* scores. The highest *hunger* and *prospective eating* scores occurred upon arrival to the lab, and the lowest *hunger* and *prospective eating* scores were found following breakfast (Fig. 5).

#### Condition

There was a significant main effect of condition on *hunger*, *fullness*, *satisfaction*, and *prospective eating* (*p* < 0.05). Participants were significantly hungrier during the Cold condition than they were during the Hot condition (Cold = 4.29 ± 1.35 vs. Hot = 3.31 ± 1.35) (p < 0.05). They felt significantly fuller and more satisfied during the Hot condition than they did during the Cold condition (Hot: *fullness* = 5.5 ± 1.81, *satisfaction* = 5.62 ± 1.77 vs. Cold: *fullness* = 4.77 ± 1.71, *satisfaction* = 4.75 ± 1.68) (p < 0.05). There was also a significant difference in how much participants felt they could eat (*prospective eating*); they felt that they could eat more during both the Sedentary and Cold conditions, as compared to the Hot condition (Sedentary = 4.74 ± 1.83; Cold = 4.74 ± 1.68 vs. Hot = 3.69 ± 1.77). There were no other significant differences between the conditions. There were also no significant interactions between time and condition for any of the appetite indices. When assessing the AUC_0-8h_ for *hunger*, *fullness*, *satisfaction*, and *prospective eating* sensation the same trends emerged (Table 4).

**Table 4:**
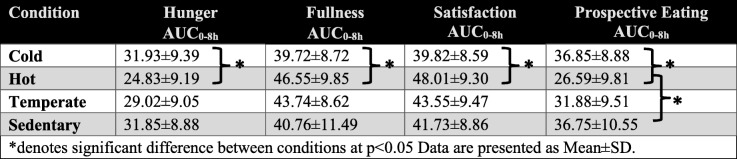
The area under the curve for the 4 indices of appetite by condition while in the environmental chamber

#### Correlations between indices of appetite sensation and food consumed

Pearson product-moment correlations were calculated among the four indices of appetite sensation (*hunger*, *fullness*, *satisfaction*, and *prospective eating*), and the amount of energy (kcal) and macronutrients (protein, carbohydrate, and fat) consumed in the 30 min following the assessment of subjective appetite. Medium positive correlations were found between *hunger* and the amount of energy (r = 0.41, *n* = 645) and nutrients (protein (g): r = 0.40, n = 645; carbohydrate (g): r = 0.39, n = 645; fat (g): r = 0.41, n = 645) consumed 30 min following appetite sensation assessment (*p* < 0.05). Similar correlations were also found between *prospective eating* and food consumed (energy (kcal: r = 0.41, n = 645); protein (g): r = 0.41, n = 645; carbohydrate (g): r = 0.40, n = 645; fat (g): r = 0.40, n = 645) (p < 0.05). On the other hand, medium negative correlations were found between *fullness* and all measures regarding energy (r = − 0.45, n = 645) and nutrient intake (protein (g): r = − 0.43, n = 645; carbohydrate (g): r = − 0.43, n = 645; fat (g): r = − 0.45, n = 645) (*p* < 0.05), as well as between *satisfaction* and the amount of energy (r = − 0.47, n = 645) and nutrients consumed (protein (g): r = − 0.45, n = 645; carbohydrate (g): r = − 0.46, n = 645; fat (g): r = − 0.47, n = 645) (*p* < 0.05).

### Appetite hormones

**Fig. 6:**
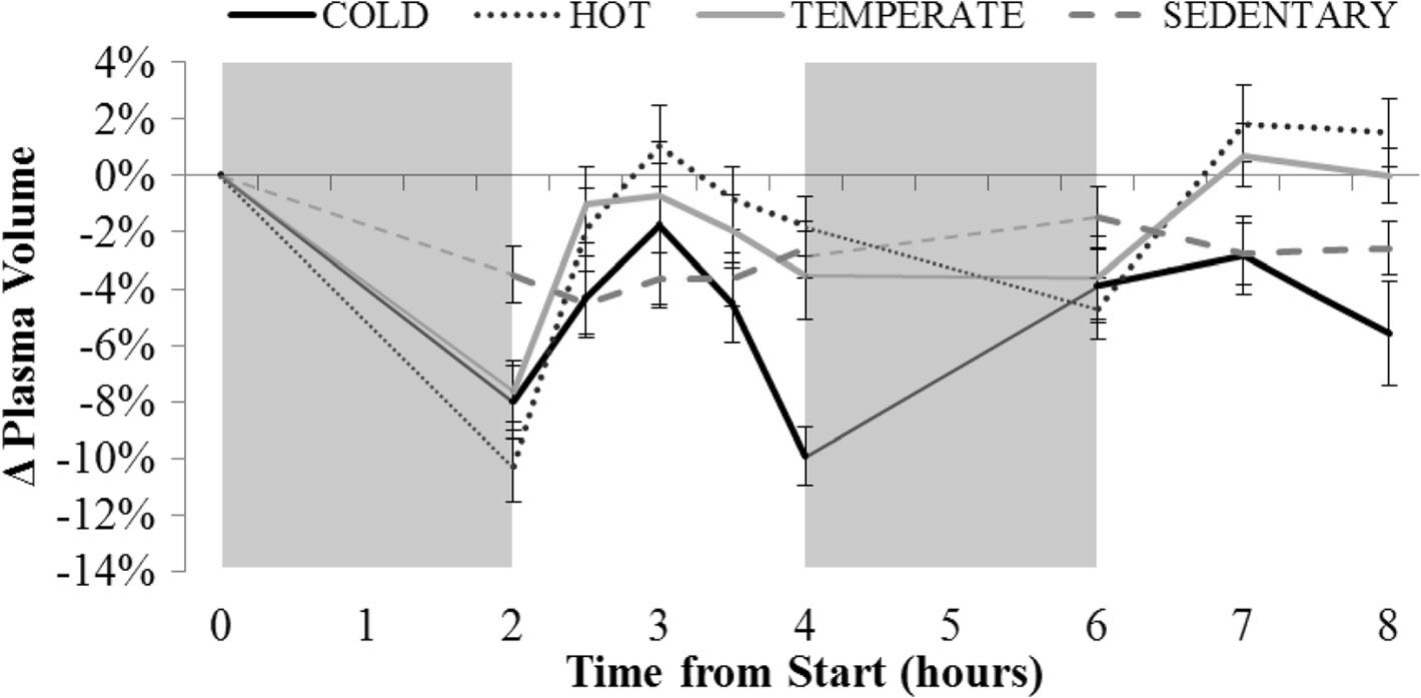
Average plasma volume change relative to fasting (time point 0) during each condition (Cold (solid black line); Hot (dotted black line); Temperate (solid grey line); Sedentary (dashed grey line)). The grey boxes represent the two-hour ‘activity blocks’, although during the Sedentary condition the participants were inactive during these blocks. The ‘0’ time point represents the fasting blood sample collected outside of the environmental chamber prior to trial commencement, all other blood samples were collected in the environmental chamber. Data are presented as mean ± SEM

All plasma concentration values were corrected for plasma volume changes [24] and for the dilution occurring from the additives added to the tubes upon sample collection. It was necessary to correct for plasma volume changes, as plasma volume varied greatly with time and condition (Fig. 6).

#### Time

##### GLP-1

**Fig. 7:**
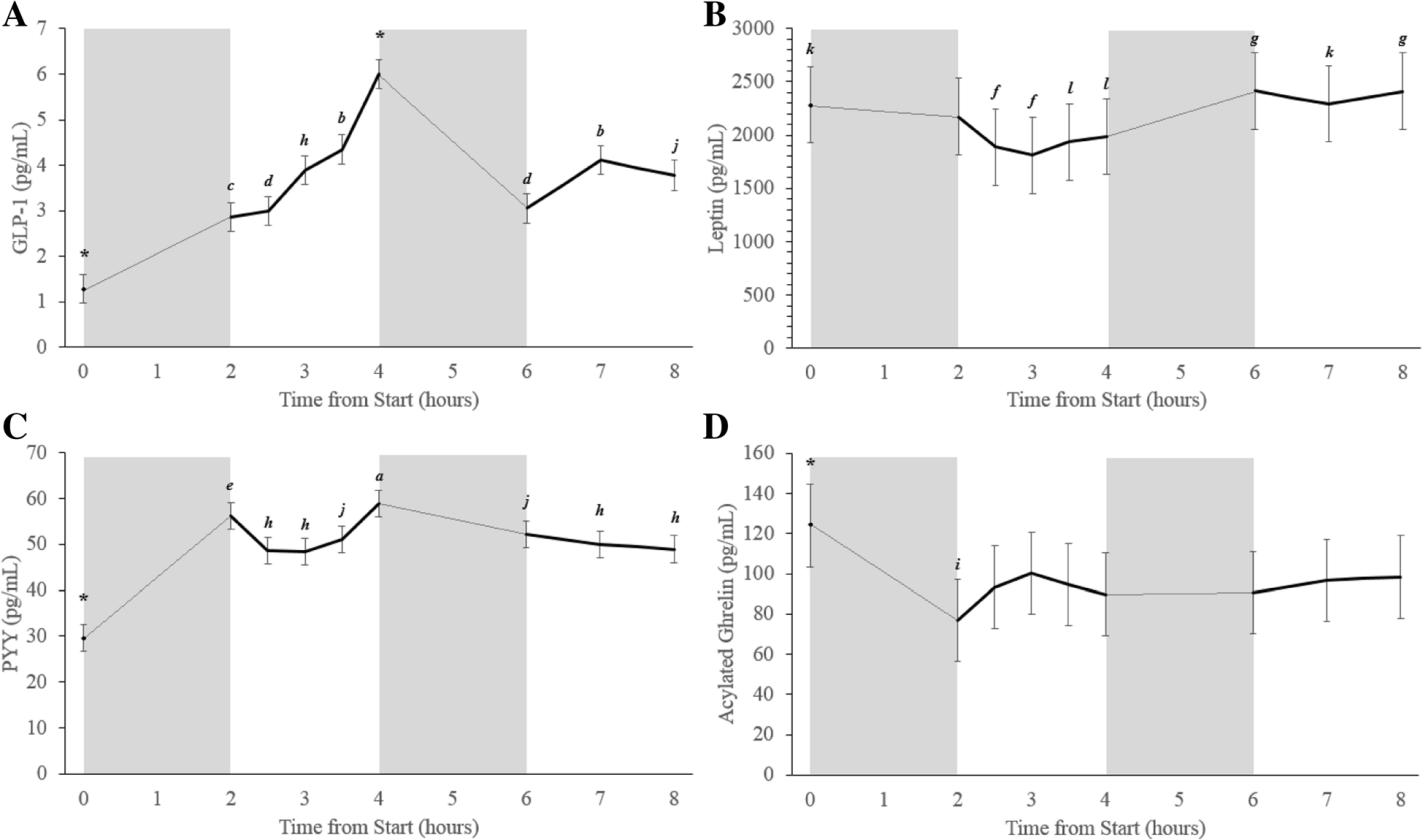
Average appetite hormone concentrations collected at each time point during all conditions. Graph A displays GLP-1 results for all participants, graph B displays Leptin results for male participants only, graph C displays PYY results for all participants, and graph D displays acylated ghrelin results for all participants. The grey boxes represent the two-hour ‘activity blocks’, although during the Sedentary condition the participants were inactive during these blocks. The ‘0’ time point represents the fasting blood sample collected outside of the environmental chamber prior to trial commencement, all other blood samples were collected in the environmental chamber. * indicates that the hormone concentrations were significantly different from all other timepoints p < 0.05. *a* indicates that the hormone concentrations were significantly different from 0, 2.5, 3, 3.5, 6, 7, and 8 h p < 0.05.*b* indicates that the hormone concentrations were significantly different from 0, 2, 2.5, 4, and 6 h p < 0.05. *c* indicates that the hormone concentrations were significantly different from 0, 3, 3.5, 4, and 7 h p < 0.05. *d* indicates that the hormone concentrations were significantly different from 0, 3.5, 4, and 7 h p < 0.05. *e* indicates that the hormone concentrations were significantly different from 0, 2.5, 3, and 8 h p < 0.05. *f* indicates that the hormone concentrations were significantly different from 0, 6, 7, and 8 h p < 0.05.*g* indicates that the hormone concentrations were significantly different from 2.5, 3, 3.5, and 4 h p < 0.05. *h* indicates that the hormone concentrations were significantly different from 0, 2, and 4 h p < 0.05. *i* indicates that the hormone concentrations were significantly different from 0, 3, and 8 h p < 0.05. *j* indicates that the hormone concentrations were significantly different from 0, and 4 h p < 0.05. *k* indicates that the hormone concentrations were significantly different from 2.5, and 3 h p < 0.05. *l* indicates that the hormone concentrations were significantly different from 6, and 8 h p < 0.05. Data are presented as mean ± SEM

GLP-1 concentrations changed quite considerably over time and several significant differences were observed (Fig. 7a). Only two time-points were significantly different from all other time-points: fasting, and 4 h post chamber entrance. GLP-1 was the lowest when the participants were fasting, and highest at 4 h following chamber entrance (immediately prior to activity block 2) (Fig. 7a) (*p* < 0.05).

##### Leptin

No significant differences were measured when assessing leptin levels over time when all (both male and female) participants were included (data not shown). However, leptin concentrations are known to be different between males and females even after accounting for fat mass differences between the sexes [57]. As a result, when the data for the 14 males was assessed separately, leptin levels were found to vary over time (Fig. 7b). When considering female data separately (*n* = 4), leptin concentrations were not different over time (data not shown).

##### PYY

PYY levels upon arrival to the lab were significantly lower than all time-points (Fig. 7c) (*p* < 0.05). Concentrations of PYY were higher at 4 h post chamber entrance, than they were at 0, 2.5, 3, 3.5, 6, 7, and 8 h following chamber entrance (p < 0.05). Figure 7c displays all other less prominent variations in PYY levels over time.

##### Acylated ghrelin

Circulating acylated ghrelin concentrations were significantly higher upon arrival to the lab than they were at any other time-point (p < 0.05) (Fig. 7d). Immediately following the first exercise block (2 h post chamber entrance), acylated ghrelin concentrations were significantly lower than they were at 6, and 8 h post entrance (p < 0.05). No other significant differences were found (Fig. 7d).

#### Condition

Regardless of whether the results were analyzed using the concentration values attained, or as a percent change from average fasting values (data not shown), the same trends emerged.

##### GLP-1

**Fig. 8:**
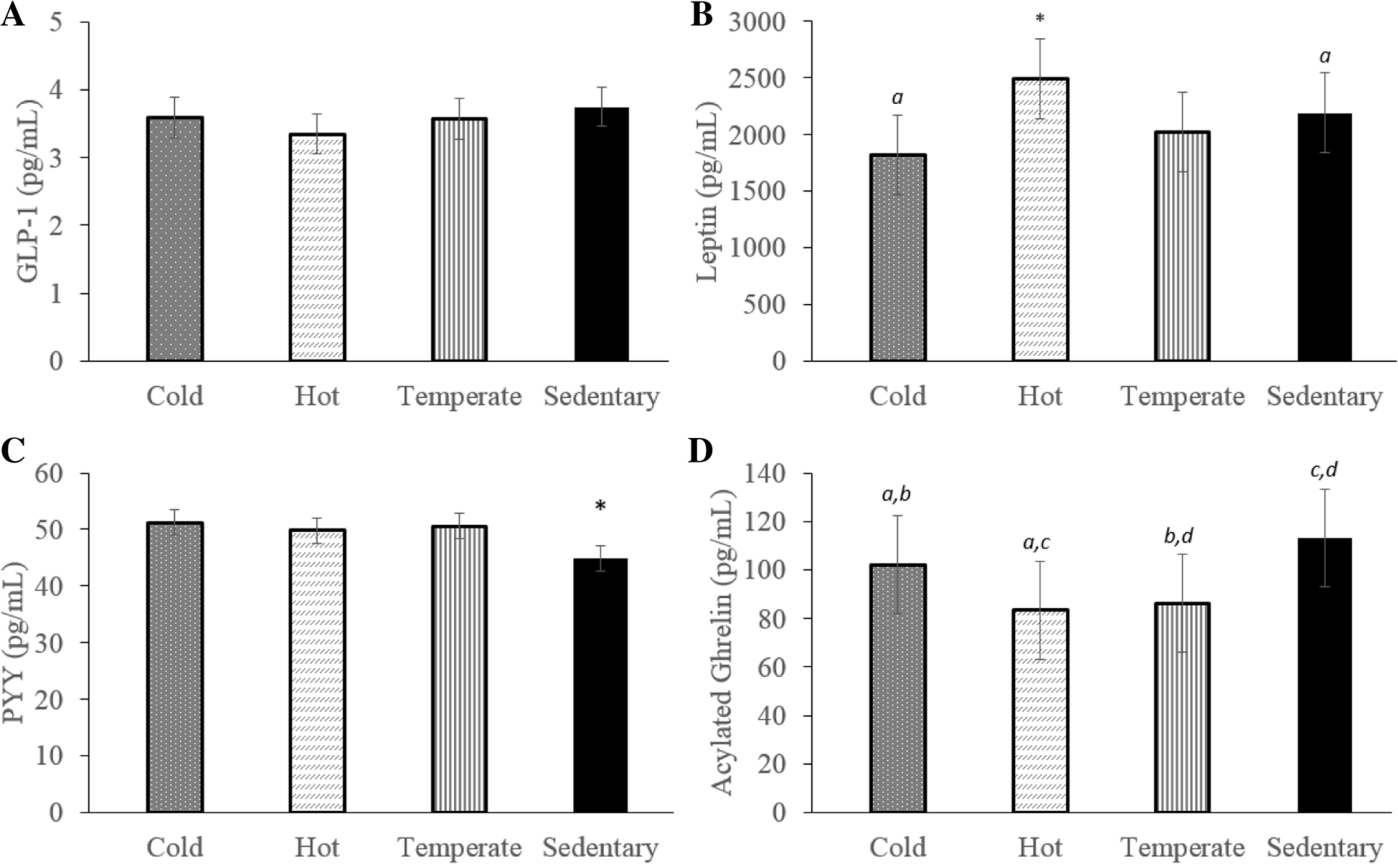
Average hormone concentrations during each condition (Cold (dotted bars); Hot (diagonally lined bars); Temperate (vertically striped bars); Sedentary (black bars)). Graph A displays GLP-1 results for all participants, graph B displays Leptin results for male participants only, graph C displays PYY results for all participants, and graph D displays acylated ghrelin results for all participants. * indicates a significant difference as compared to ALL other conditions p < 0.05. Bars labelled by the same letter (*a, b, c, d*) on the graph are significantly different from each other p < 0.05. Data are presented as mean ± SEM

No significant differences between conditions were observed for GLP-1 (Fig. 8a).

##### Leptin

No significant differences between conditions were measured for leptin when data for men and women was analyzed together (data not shown). When analyzed separately, leptin concentrations in males were significantly higher during the Hot condition (2494.1 ± 351.6 pg/mL) as compared to all other conditions: Cold (1825.8 ± 352.0 pg/mL), Temperate (2016.0 ± 352.2 pg/mL), or Sedentary (2192.4 ± 351.6 pg/mL) (p < 0.05). In males, leptin concentrations were also significantly higher during the Sedentary condition vs. the Cold condition (Fig. 8b) (p < 0.05). When considering female data separately, leptin concentrations were not found to be significantly different between conditions (data not shown); this is not surprising due to the low number of female participants.

##### PYY

Circulating PYY concentrations were significantly lower during the Sedentary condition (44.9 ± 2.3 pg/mL) as compared to all other conditions: Cold (51.3 ± 2.3 pg/mL), Temperate (50.6 ± 2.3 pg/mL), or Hot (49.8 ± 2.3 pg/mL) (p < 0.05). No other significant differences were found (Fig. 8c).

##### Acylated ghrelin

Acylated ghrelin levels were higher during both the Sedentary (113.1 ± 20.2 pg/mL) and Cold (102.2 ± 20.2 pg/mL) conditions than they were during either the Hot (83.4 ± 20.2 pg/mL) or Temperate (86.3 ± 20.2 pg/mL) conditions (p < 0.05). No significant differences were observed between Sedentary and Cold conditions, or between Hot and Temperate conditions (Fig. 8d) (*p* > 0.05).

#### AUC

Acylated ghrelin concentrations were significantly lower during the Temperate (41,234 ± 35,534 pg/mL) condition than during either the Cold (49,740 ± 42,029 pg/mL) or Sedentary (55,964 ± 39,700 pg/mL) conditions (*p* < 0.05). No significant differences were found between conditions for the AUC_0-8h_ for any other appetite hormone.

#### Correlations between hormones

As expected, fasting leptin levels were significantly higher in females (p < 0.05) and were significantly correlated to body fat percentage (r = 0.75, *n* = 14, *p* < 0.05) but not body mass. No other hormones were correlated with any of the measured body stature or composition variables. When looking at correlations between the hormones of interest, there was a small but significant positive correlation between PYY and GLP-1 (r = 0.25, *n* = 571, p < 0.05). Similarly a small positive correlation between acylated ghrelin and leptin (r = 0.11, *n* = 577, *p* < 0.05) was found.

#### Correlations between hormones and food consumed

Pearson product-moment correlations were determined between the concentrations of the measured hormones and the amount of food (kcal) and nutrients (protein, carbohydrate, and fat) consumed in 30 min following the blood sample collection. Table 5 demonstrates that there were only weak, albeit significant, correlations among some of the appetite hormones and the total energy and macronutrients consumed.

**Table 5:** Pearson product-moment correlations between appetite hormones and dietary consumption 30 min following sample collection

	PYY (pg/mL)	GLP-1 (pg/mL)	Leptin (pg/mL)	Acylated Ghrelin(pg/mL)
Amount of energy consumed (kcal)	−0.25^a^ (530)	−0.29^a^(530)	0.04(530)	0.08(519)
Protein (g)	−0.24^a^(530)	−0.28^a^(530)	0.02(530)	0.09^a^(519)
Carbohydrate (g)	− 0.23^a^(530)	−0.28^a^(530)	0.04(530)	0.06(519)
Fat (g)	−0.25^a^(530)	−0.28^a^(530)	0.03(530)	0.10^a^(519)

Pearson’s r (n)

^a^signifies significant correlation p < 0.05

#### Correlations between hormones and indices of appetite sensation

Similarly, when assessing the correlations between the appetite hormones and the different aspects of appetite sensation, weak correlations were detected between the hormones of interest and the four indices of appetite perception (Table 6).

**Table 6:** Pearson product-moment correlations between appetite hormones and appetite sensation

	PYY (pg/mL)	GLP-1 (pg/mL)	Leptin(pg/mL)	Acylated Ghrelin(pg/mL)
Hunger	−0.24^a^(455)	−0.28^a^(455)	−0.05(455)	0.07(447)
Fullness	0.24^a^(455)	0.22^a^(455)	−0.30^a^(455)	−0.13^a^(447)
Satisfaction	0.26^a^(455)	0.23^a^(455)	−0.25^a^(455)	−0.15^a^(447)
Prospective Eating	−0.22^a^(455)	−0.23^a^(455)	0.16^a^(455)	0.08(447) *p* = 0.077

Pearson’s r (n)

^a^signifies significant correlation p < 0.05

## Discussion

The primary objective of this study was to determine the impact of arduous physical activity in varying ambient temperatures on appetite regulating hormone concentrations, subjective appetite sensations, and dietary intake. Although hormonal responses pointed towards appetite suppression with exercise (with a partial blunting of that response in the cold), and subjective appetite sensation results suggested that appetite was highest in the cold, and lowest in the heat, actual dietary intake was the same regardless of whether the condition was sedentary or active and also regardless of the variations in ambient temperature.

### Dietary intake

Interestingly, a substantial amount (4 h) of arduous exercise (expending > 1200 kcal above resting), even when completed in harsh ambient temperatures (− 10 °C, and 30 °C) did not alter dietary intake (both EI and macronutrient intake).

Although previous studies have also reported no effect of acute exercise on subsequent EI, [10, 69], their exercise protocols were of significantly shorter duration (< 2 h). In order to stimulate EI above resting levels, some have previously suggested that high volumes of physical activity (≥2 h) [26] and/or arduous exercise [55] are necessary. To our knowledge the participants in this study underwent the largest exercise stimulus (completing 4 h of physical activity and expending > 1200 kcal above resting) compared to other published studies of appetite / exercise interactions, and yet measurements of 24 h EI were remarkably similar among conditions (Sedentary intake: 2924 ± 1066 kcal vs. Temperate intake: 2923 ± 802 kcal).

Ambient temperature alone is thought to impact EI leading to increased food consumption as ambient temperature decreases and vice versa [13, 41]. The effect of exercise in various temperature conditions on dietary intake has also been found to follow this trend [22, 71]. [22] found that overweight participants ate significantly more if their brisk walk was conducted in a cold, vs. temperate environment, and Wasse et al. [71] found trends towards both lower EIs in the heat, and higher EIs in the cold. However, EI in the present study was unchanged by ambient temperature. Even when EI was classified as 1) typical energy intake (defined as intake within 10% of a participant’s median intake), 2) low energy intake (consumption below 10% of a participant’s median intake), and 3) high energy intake (consumption above 10% of a participant’s median intake); no significant differences between conditions were found; roughly half of the participants consumed within 10% of their median intake in each condition, approximately a quarter had a “low energy intake” in each condition, and the remaining quarter had a “high energy intake” in each condition.

In terms of energy balance, our study supports several other reports that acute exercise results in an acute negative energy balance [51, 67–69] in participants who have ad libitum access to food after exercise. This was the case in the current study even though our 24 h EE estimates may have underestimated true EE (as the participants may have slept for less than 8 h, and they may have been more active after leaving the chamber than they were in the chamber on the Sedentary day). The negative energy balance detected in the active conditions (Hot, Temperate, and Cold) was likely greater than estimated, and our conservative EE assessment likely underestimated the active conditions the most, since post-exercise elevations in oxygen consumption were not included in the estimation. Additionally, ‘rest’ in the cold likely would have resulted in EE above that seen in a temperate or warm environment, as even mild cold (feeling “cool but not too cold”) has previously been found to increase resting metabolism [23]. In the current study, core temperature was well defended in the Cold (not dropping below 36 °C at any time in any participant), however 1/3 of the participants reported being “cold” or “very cold” at some point during the Cold trial, and researchers observed mild shivering in some participants, suggesting that EE during rest in the Cold condition was likely underestimated.

Notwithstanding these limitations, most of our participants (15 of 18) were in a positive energy balance during the Sedentary condition and a negative energy balance (11 of 18) throughout all three active conditions (Hot, Temperate, and Cold).

It is important to note, that according to our food satisfaction surveys [38], the military rations were deemed to be ‘acceptable’ in a variety of different categories including: ease of preparation/consumption, taste, texture, saltiness, sweetness, density/fullness, digestibility and overall adequacy. Therefore, it is unlikely that the military rations were over or under consumed based on the palatability or lack thereof of the provided food. Additionally in terms of the total amount of food consumed, neither the food available (being limited to military rations vs. consuming foods usually available to them), nor the surrounding environment (at home vs. in the environmental chamber) had any effect: participants consumed the same amount of food regardless of whether they consumed military rations or their own food at home, and they consumed the same amount of energy and macronutrients two days before the chamber day (at home) as they consumed on each day in the environmental chamber [3, 38].

### Subjective response

In contrast with previous studies [15, 44, 69], in the current investigation no aspect of subjective appetite was significantly affected by exercise alone (no differences were found between Sedentary and Temperate conditions). However, unlike most other studies of appetite, the current study’s participants could eat ad libitum throughout the conditions; consequently our participants ate whenever it suited them, thereby increasing the inter-subject variation among the appetite scores assessed at each time point.

When evaluating the impact of ambient temperature, exercise in the heat suppressed subjective appetite more than during the Cold condition; for *prospective eating*, the suppression was evident when compared to the Sedentary condition as well. This is in agreement with Kojima et al. [46] who reported that *hunger* and *motivation to eat* were suppressed to a greater extent at 36 °C and 24 °C than they were at 12 °C, while Wasse et al. [71] found suppressed *hunger* and *prospective eating* scores at 30 °C as compared to 20 °C, and lower *satisfaction* and *fullness* scores at 10 °C as compared to 20 °C. Consistent with these studies, the current study found a similar trend towards subjective appetite increasing in the cold, and diminishing in the heat.

### Hormonal response

Neither exercise alone, or in combination with a temperature challenge had any effect on circulating GLP-1 levels. Alternatively, exercise and ambient temperature induced hormonal changes in the direction expected to suppress appetite for both acylated ghrelin and PYY; exercise in both temperate and hot environments suppressed acylated ghrelin concentrations, while exercise in all temperature conditions increased circulating PYY levels.

Although large changes in energy balance due to heavy exercise (e.g. marathon running) has previously been found to decrease leptin concentrations [48], leptin levels in the present study were not affected by exercise alone. Leptin is not typically considered to be involved in short-term appetite regulation with most studies finding no effect of exercise on circulating leptin levels [21, 69]. Interestingly, cold exposure has previously been found to decrease plasma leptin levels [56, 74], and while no significant results were observed when considering all of the participants data together, analyzing only the male data in the current study yielded different results. Leptin concentrations in males were significantly lower in the Cold condition vs. the Sedentary and Hot conditions, but there was no significant difference between the Cold and Temperate conditions. Leptin concentrations were analyzed separately in males and females since leptin concentrations are known to be very different between the sexes [57], and only four of the 18 participants who completed the study were females. Leptin concentrations were not different between the Cold and Temperate conditions suggesting that the small (0.04 °C) but significant decrease in average core temperature between the Temperate and Cold conditions (data not shown) was likely not sufficient to suppress circulating leptin levels. This is not surprising since our participants were active for 4 of the 8 h they spent in the − 10 °C environmental chamber, and they were dressed appropriately for the environmental condition.

Interestingly, leptin was negatively correlated with *fullness* and *satisfaction*, and positively correlated with *prospective eating*. While most previous studies have shown no relationship between blood leptin levels and subjective appetite sensations [40, 43], positive correlations between leptin concentrations and satiety have been found following weight loss [34]. Whether previous exercise modulates this relationship is currently unclear considering that Tsofliou et al. [64] found positive correlations between leptin and both *fullness* and *satiety* and negative correlations between leptin and both *hunger* and *desire to eat* while Vatansever-Ozen et al. [69] found no correlation between circulating leptin concentrations and any factor of subjective appetite following exercise. It is possible that the conflicting results are due to methodological differences employed in the different studies; in particular, the present study was the only one that did not schedule specific meal times, and perhaps as a result, the relationship between leptin and subjective appetite may have been modified compared with those studies where meals were not ad libitum and were scheduled for specific times.

**Fig. 9:**
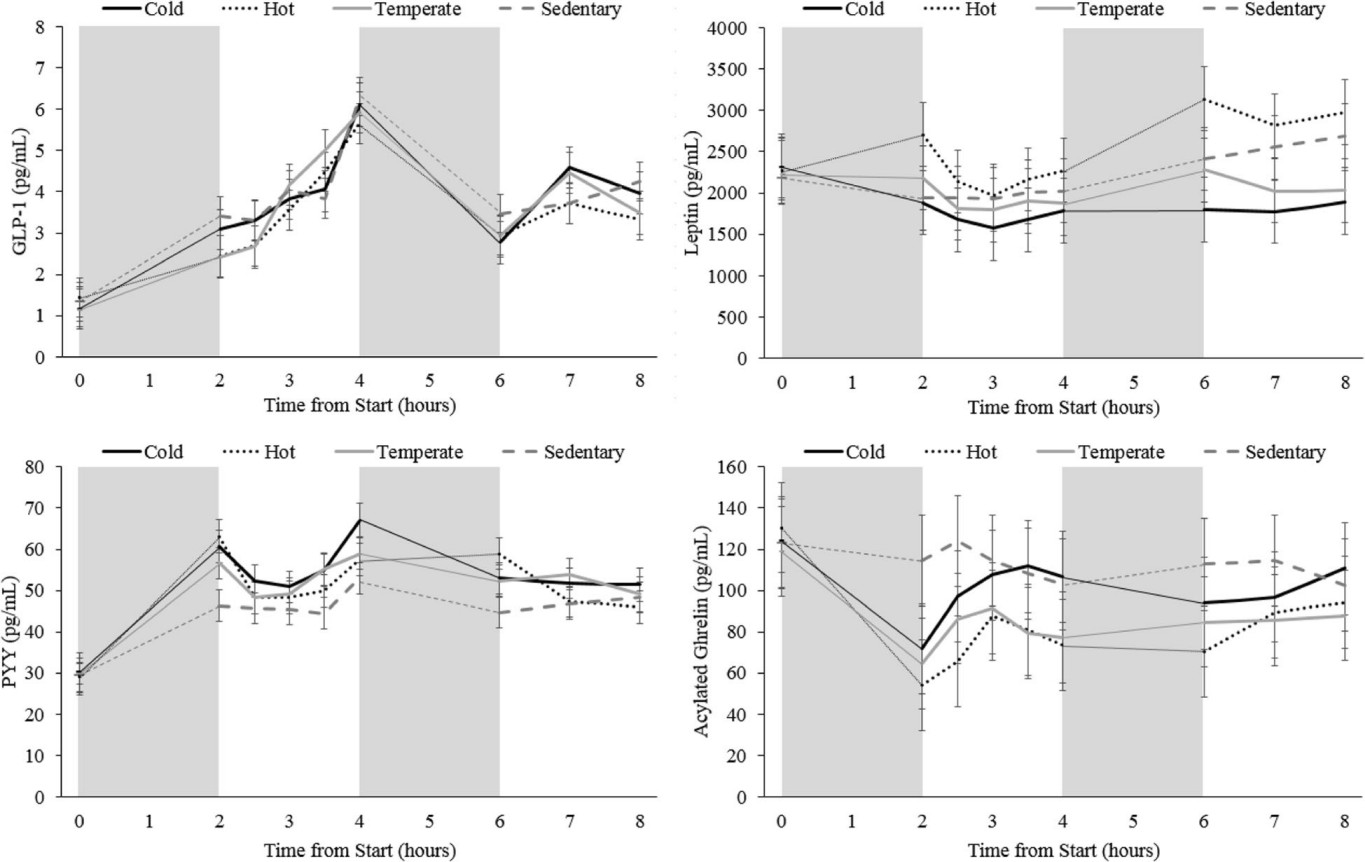
Average appetite hormone concentrations collected at each time point during each condition (Cold (solid black line); Hot (dotted black line); Temperate (solid grey line); Sedentary (dashed grey line). Graph A displays GLP-1 results for all participants, graph B displays Leptin results for male participants only, graph C displays PYY results for all participants, and graph D displays acylated ghrelin results for all participants. The grey boxes represent the two-hour ‘activity blocks’, although during the Sedentary condition the participants were inactive during these blocks. The ‘0’ time point represents the fasting blood sample collected outside of the environmental chamber prior to trial commencement, all other blood samples were collected in the environmental chamber. Data are presented as mean ± SEM

Regarding GLP-1, most studies suggest that circulating GLP-1 levels increase following exercise [36, 51, 66, 67], while one study found decreases in GLP-1 following 40 min of moderate intensity walking in overweight/obese women [68]. In the present study, circulating GLP-1 was not significantly changed by exercise or ambient temperature. In contrast with other studies when specific meal times were scheduled, participants in the present study were able to eat at any time; this resulted in large variations in GLP-1 at every time point. As a result, it is likely that any effect of exercise, or exercise in combination with ambient temperature was masked by the large inter-individual and inter-sample variations (Fig. 9). Regardless of the large inter-individual variations, GLP-1 was still found to be positively correlated with both *fullness* and *satisfaction* and negatively correlated with *hunger* and *prospective eating*. Although GLP-1 is not always found to be correlated with subjective appetite [20], the results in the current study are agreement with many other works [25, 65].

In the present study, circulating PYY concentrations were increased similarly in the Temperate, Hot, and Cold conditions. Exercise typically increases circulating PYY levels [36, 46, 51, 66, 67], and this increase is thought to be independent of ambient temperature [22, 46]. The current study is in agreement with these previously reported observations. Correlations between PYY and subjective appetite were also found in the expected directions. PYY was positively correlated with both *fullness* and *satisfaction* and negatively correlated with *hunger* and *prospective eating*. While PYY is considered to be a satiety hormone, most previous research has not found PYY to be correlated with any aspect of appetite sensation [15, 25, 33, 45]. The significant correlations found in the current study are possibly due to the fact that the participants in our study were able to eat whenever it suited them.

The first exercise bout decreased circulating acylated ghrelin concentrations in the present study, a finding which is consistent with earlier reports [17, 44, 69, 71]. Ambient temperature has previously been reported to impact total ghrelin concentrations by increasing plasma ghrelin levels in the cold (2 °C), and decreasing them in the heat (30 °C), as compared to levels found at 20 °C [63]. However, the interactions of ambient temperature, exercise, and ghrelin are equivocal. While, some studies found no effect of ambient temperature on acylated ghrelin concentration when exercise is performed at temperatures ranging from 10 °C to 36 °C [46, 71], Crabtree et al.[22] found higher acylated ghrelin levels following brisk walking in the cold (8 °C), as compared to a neutral environment (20 °C). Consistent with the findings of Crabtree et al., the present study found that the acylated ghrelin suppression observed with exercise is blunted in the cold. However, in the current study exercise in 30 °C did not result in a suppression of acylated ghrelin beyond the levels observed with exercise at a comfortable temperature. The orexigenic hormone acylated ghrelin was found to be negatively correlated with *fullness* and *satisfaction*, yet was not correlated with either hunger or prospective eating. While many appetite hormones are thought to impact appetite sensation, the correlations between these variables are not often observed [16, 25, 33].

Overall exercise altered the hormonal milieu in a manner expected to suppress appetite, and ambient temperature only had minor additional effects beyond those associated with exercise alone.

Although food accessibility during the measurement periods likely affected the timing of meals (due to the oro-nasal mask), variations in: ambient temperature, exercise, hormone concentrations, and subjective appetite sensation had no effect on the amount and timing of food intake, suggesting that none of these factors systematically change eating patterns in any predictable way. Dietary intake in our population was surprisingly similar across conditions. Interestingly, participants were generally unaware of how much they ate; when asked whether they thought they consumed more or less than usual, participants were only correct 35% of the time, 46% of the time they thought they ate less than they did, and 19% of the time they thought they ate more than they did. Finally, while menstrual cycle phase was documented in the present study, it was not controlled for, and the low number of women in the study prevented any further investigation into how the menstrual cycle may have impacted the results.

It is likely that there are other factors not assessed in the current investigation that impacted appetite, such as “expected satiety” [37] (the learned association between a familiar food and its satiating effect which allows an individual to anticipate how much of that food they should eat to feel satiated). The current investigation focused on physical and physiological aspects of appetite and to the best of our knowledge this is the first controlled laboratory investigation to simulate a strenuous 8-h work day in varying environmental temperatures and simultaneously evaluate appetite, hormonal concentrations, dietary intake, and EE. It is our belief that investigations such as the current one, with fewer limitations on food intake compared to other studies, has stronger external validity with regard to the hormonal patterns, and demonstrates that: 1) wide variations in physical activity impact PYY, while exercise and environmental temperature impact, leptin, and acylated ghrelin concentrations significantly enough that differences between conditions are evident even when participants are consuming food at different times; 2) exercise in the heat and cold result in significantly different appetite sensations – subjective appetite is significantly lower when exercising in the heat as compared to the cold; 3) irrespective of 1 and 2, dietary intake remains remarkably similar regardless of condition suggesting that variations in hormone concentrations and subjective appetite have minimal effect on dietary intake.

## Abbreviations

AEBSF: 4-(2-Aminoethyl) benzenesulfonyl fluoride hydrochloride
ANOVA: Analysis of variance
AUC: Area under the curve
CAF: Canadian Armed Forces
ECLIA: Electrochemiluminescent immunoassays
EE: Energy expenditure
EI: Energy intake
GLP-1: Glucagon-like peptide-1
GSQS: Groningen Sleep Quality Scale
MSD: Meso Scale Diagnostics
PSQI: Pittsburgh Sleep Quality Index
PYY: Peptide YY
REI: Relative energy intake
RH: Relative humidity
VASA: Visual analogue scales for appetite
*V̇O*_*2peak*_: Peak aerobic power
